# Complementary Feeding in the Clinical Practice: An Exploratory Survey among Italian Primary Care Pediatricians

**DOI:** 10.3390/nu16183127

**Published:** 2024-09-16

**Authors:** Maria Elena Capra, Nicola Mattia Decarolis, Brigida Stanyevic, Antonella Giudice, Delia Monopoli, Cosimo Neglia, Greta Ramundo, Susanna Esposito, Giacomo Biasucci

**Affiliations:** 1 Pediatrics and Neonatology Unit, Guglielmo da Saliceto Hospital, 29121 Piacenza, Italy; m.capra@ausl.pc.it (M.E.C.); g.biasucci@ausl.pc.it (G.B.); 2 Pediatric Clinic, Department of Medicine and Surgery, University of Parma, 43126 Parma, Italy; 3 Department of Medicine and Surgery, University of Parma, 43126 Parma, Italy

**Keywords:** complementary feeding, survey, primary care pediatrician

## Abstract

**Introduction:** Complementary feeding (CF) is the process of introducing solid or liquid foods (complementary foods, CFs) other than human breast milk (HBM) or infant formula into infants’ diet when HBM or infant formula is no longer sufficient to meet infants’ nutritional needs. Primary care pediatricians (PCPs) are paramount in guiding and educating infants’ families during CF. **Materials and Methods:** Our exploratory survey aimed to investigate PCPs’ current clinical approach to managing CF. From 1 March 2024 to 30 April 2024, a digital questionnaire composed of 32 multiple-choice questions investigating PCPs’ attitudes toward CF in healthy, full-term infants was proposed to 1620 PCPs contacted through scientific societies. **Results:** The questionnaire was completed voluntarily; 707 PCPs (79.5% female, 66.1% aged over 50 years) fully responded to the survey in the proposed timeframe (participation rate 43.6%). Among the responders, 47.5% recommended traditional CF; 42.1% declared to know the baby-led weaning (BLW) approach and on-demand CF (ODCF), but only 32.8% and 12.5% of them recommended these types of CF, respectively. The vast majority (95%) of participants recommended that CF start between 4 to 6 completed months of age. CF routinely based on vegetarian or plant-based diets was supported by 45/707 (6.1%), only if planned by a specialist by 253/707 (35.8%), and only upon request by caregivers by 257/707 (36.3%). Egg and fish introduction was mostly advised in the first year of life, although in case of a positive family history of food allergy, 13.3% of participants recommended the introduction of egg and fish after 12 months. **Conclusions:** In conclusion, PCPs did not display a homogenous approach to CF; further studies and educational programs are needed to achieve more flexibility and knowledge on this important nutritional issue.

## 1. Introduction

### 1.1. Complementary Feeding: The State of the Art

The World Health Organization (WHO) defines complementary feeding (CF) as “the process of introducing solid or liquid foods other than human breast milk (HBM) or infant formula, known as complementary foods (CFs), into the human diet when HBM or infant formula is no longer sufficient to meet the nutritional needs of infants” [1]. The United Nations Children’s Fund (UNICEF) and the WHO have recognized appropriate CF and breastfeeding (BF) as key health-promoting practices during infants’ first two years of life [2]. As the period from conception to 24 months of age (the first 1000 days of life) is crucial for healthy growth and neurodevelopment in children, the introduction of CFs during this timeframe must be properly timed and selected to ensure positive outcomes [3,4]. The WHO recommends CF start at 6 months of age, along with BF or formula feeding (for mothers who cannot breastfeed) [5], and so does the Italian Society of Pediatrics [6], whereas both the American Academy of Pediatrics (AAP) and the European Society of Paediatric Gastroenterology, Hepatology, and Nutrition (ESPGHAN) suggest that CF should be started from 4 completed to 6 completed months of age, depending on the infant’s growth parameters, the achievement of appropriate developmental milestones, and the availability of safe CFs. Despite extending the time window for CF [7,8], both societies specifically state that the introduction of CF is not recommended before 4 months or after 6 completed months of age [7]. There is also a consensus between the different Institutions on the recommendations regarding the introduction of CFs with higher potential allergenic properties, added salt, added sugar, and gluten. Although the most suitable time window for the introduction of CFs with higher potential allergenic properties is still not universally agreed upon [9], current evidence is in favor of introducing most CFs with higher potential allergenic properties between 6 and 12 completed months of age, possibly during BF [7,8,10,11,12]. Due to several factors, including low iron bioavailability and potential intestinal micro-hemorrhages, ESPGHAN and AAP recommend against the introduction of whole cow’s milk until 12 completed months of age [7,8]. In line with this, several Italian pediatric societies (SIPPS, FIMP, SIDOHaD, and SINUPE) recommend delaying cow’s milk introduction even after 24 months of age [6]. Most organizations also recommend not to add sugar and/or salt to home-made meals for children [7,13] during the first year of life and to limit their intake at least during the first 24 months of life [14] and possibly also in early childhood [6]. Currently, the available evidence indicates that the timing of the gluten introduction has no risk-increasing or preventing effect on the development of celiac disease (CD). Accordingly, SIPPS, FIMP, SIDOHaD, and SINUPE recommend gluten introduction at the beginning of the CF period, whereas ESPGHAN suggests a wider time window, i.e., between 4 and 12 months [6,7]. 

Many scientific societies worldwide are engaged in providing the best possible recommendations for children’s health, and they and healthcare professionals face other challenges. Indeed, new dietary models are gaining popularity among the general population, as demonstrated by the remarkably increasing prevalence of people following a plant-based diet (PBD) in recent decades. Italian data from a survey conducted by Eurispes in 2023 show that the percentage of people following a vegetarian and vegan diet is 4.2% and 2.4%, respectively [15]. Consequently, we should also expect a potentially increasing number of children following such dietary models; despite this, there is still no consensus among pediatric scientific societies on the effects of these dietary models on children’s health, due to the lack of robust data [16]. According to the Italian Position Paper on Vegetarian Diets in Pregnancy and Developmental Age, there is still insufficient scientific evidence to determine at which age it is safe to start a vegetarian diet [17]. Other organizations state that a well-balanced vegetarian diet is appropriate for people of all ages, including infancy [18,19,20,21], whereas ESPGHAN does not recommend a vegan diet during the first two years of life [7].

### 1.2. Primary Care Pediatrician and Approach to Complementary Feeding

In Italy, all children are provided with a primary care pediatrician (PCP) by the Italian National Healthcare System. This professional has an institutionalized role in the care of pediatric patients over time, with a primary focus on general health, growth, and, inevitably, nutrition and diet. When approaching the CF period, primary care pediatricians should adequately inform infants’ parents and caregivers about the dietary pattern to be adopted. Nowadays, several different approaches to CF are possible: Caregivers may prepare CFs themselves, using fresh ingredients in the form of spoon-fed pureed foods, spoon-fed lumpy foods, finger foods, and beverages, or they may purchase industrially processed, ready-made foods with the recommended balance of nutrients appropriate for this age group, known as “baby foods” (BFs) [22]. “Traditional weaning” (TW), “traditional spoon feeding”, and “standard weaning” or “parent-led weaning” are extensively endorsed by the scientific literature consensus. With these approaches, the child is mostly passive, receiving food in the form of cream/puree using a spoon. In contrast, with the baby-led weaning (BLW) approach [23], infants are offered pieces of food in a size and shape (typically stick-shaped) that they can pick up and take by themselves; parents are responsible for what to offer, but babies decide what to eat, how much, and how quickly. In another approach proposed by the Italian pediatrician Lucio Piermarini and known as “on-demand complementary feeding” (ODCF) or “self-weaning” (SW) [24], infants are offered minced and mashed food, which is taken with a spoon. The mode, timing, quantity, and consistency of food offered are based on the infants’ level of neuro-psycho-motor and physical development. Unlike TW, in BLW and ODCF, the foods given to the infants are partly the same as those taken by caregivers [4]. The same can be said about the so-called “traditional spoon-feeding with adult food tastings” approach, which, while following the TW style, allows parents to let their children taste any mashed and minced foods when sharing a meal, somehow reproducing typical aspects of BLW or ODCF. In this sense, the role of the PCPs is once again of utmost importance, as they are responsible for the education about the importance of healthy eating for the whole family and not just for the infant.

In recent years, two 4-question surveys were conducted in Italy by the same research group between January 2015 and December 2017 (n° PCPs = 665) and between January 2022 and October 2022 (n° PCPs = 595), respectively, to capture the then-current attitude of PCPs towards CF [25,26]. In 2022, a significant reduction in the use of baby foods and the adoption of TW and an increase in the adoption of BLW and traditional spoon-feeding with adult food tastings were reported across the country. 

In this context, the aim of our exploratory survey is to investigate Italian PCPs’ current clinical approach in CF management and recommendations.

## 2. Materials and Methods

A digital questionnaire composed of 32 multiple-choice questions investigating PCPs’ attitudes toward CF in healthy, full-term infants was proposed to PCPs. We designed a specific questionnaire to investigate the timing of CF start, the known and recommended models of CF, methods to instruct parents or caregivers on CF, and attitude towards allergenic food introduction. The survey was transmitted to 1620 PCPs contacted through regional professional societies or following direct contact, over a period from 1 March 2024 to 30 April 2024. The questionnaire was completed voluntarily. In the first week of March 2024, PCPs were requested to participate in a survey on CF by an e-mail message that provided a direct link to a Google Form on Google Drive that contained the questionnaire; all the questions required mandatory answers; otherwise, the questionnaire could not be submitted correctly. A second request to participate was sent at the end of March 2024, and the collection of data from the questionnaire was finished on 30 April 2024. At the time of invitation, 1620 PCPs of a total of approximately 7000 PCPs [27] were included, which corresponds to more than 20% of the total of PCPs in Italy. Members who participated in the survey were representative of PCPs in terms of age and gender. The questionnaires were completed anonymously, and the participant age range and gender were recorded. It was not possible to complete the form more than once. The participation rate was 43.6% (707 on 1620).

The questionnaire consisted of 32 multiple-choice questions. The questionnaire items and possible response options are reported in Appendix A. All the questions were selected by a scientific ad hoc board of the SIPPs; the questions were intended to be representative of PCPs’ clinical approach of providing recommendations for CF, based on personal beliefs and scientific evidence, and to be written in the simplest way to improve response rates.

The questions concerned sources of learning about CF; the suggested age for the introduction of CF; knowledge about TW, BLW, and ODCF approaches; the CF approach usually recommended; the parents’ preferences about the CF approach to be taken; the percentage of parents following the given indications; the PCPs’ motivations for starting; anticipating or postponing the CF introduction; the dietary patterns suggested to patients of foreign ethnicity; whether or not an interview is organized with the parents to discuss the recommendations given about CF; whether they are provided with written information; whether PCPs usually recommend “baby foods”; the attitude towards PBDs; which food class they usually recommend as a first CF; and the timing of introduction of CFs with higher potential allergenic properties, gluten, soy, and added salt and sugar. The full questionnaire can be found in the Appendix A.

According to estimates by the Italian National Institute of Statistics (ISTAT), the number of PCPs working in Italy was 6962 on 30 May 2024 [27]. To obtain a representative sample with a confidence interval of 95% and a margin of error of 5%, the minimum number of respondents was calculated to be 348.1 PCPs. 

Statistical analysis was performed by using STATA^®^ software (version 12, College Station, TX, USA). All answers to the questions were reported as frequency and percentage. For multiple choice, percentages were calculated for each answer on the total number of interviewees. Comparisons between groups were performed by using the chi-square test (for frequencies equal to or greater than 5) and Fisher’s exact test (used for frequencies less than 5). Both tests were calculated on each single answer. A *p*-value below 5% (*p* = 0.05) was considered statistically significant.

## 3. Results

A total of 707 PCPs (79.5% females, 33.9% younger than 50 years old) fully responded to the survey in the given timeframe; 111/707 (15.7%) subjects were younger than 40 years old, 129/707 (18.2%) were aged between 40 and 50 years, 153/707 (21.6%) were aged between 50 and 60 years, and 314/707 (44.4%) subjects were over 60 years old. 

### 3.1. Informative Sources on CF

Overall, 439/707 (62.1%) of the participants declared that they had been informed about CF in specific refresh courses, 324/707 (45.8%) during university studies, 315/707 (44.5%) through scientific journals, 306/707 (43.3%) by consulting journals or position papers, and 95/707 (13.4%) through empirical methodology. The analysis of responses, stratified by the age of participants, revealed a significantly greater tendency among those over 50 to learn useful notions through specific refresh courses (68.1% vs. 50.4%, *p*-value < 0.001), while among those under 50, there is a greater tendency to learn through guidelines/position papers (52.9% vs. 38.3%, *p*-value < 0.001) and empirical methodology (17.9% vs. 11.1%, *p*-value < 0.01). 

### 3.2. Timing to Start CF

When asked which determinants they consider in starting CF, 21.1% (149/707) of participants indicated infant’s achievement of neurodevelopmental milestones, 15.8% (112/707) nutritional needs, 9.9% (70/707) family compliance, and 2.7% (19/707) weight gain of the infant. However, when considered as a whole, 75.2% (532/707) of the respondents considered all these aspects to be relevant to the decision. Analyzing results according to participants’ age, achieving neurodevelopmental milestones and adequate family compliance were significantly more relevant in the group of those under 50 years of age (32.1% vs. 15.4% *p*-value < 0.001 and 14.6% vs. 7.5% *p*-value < 0.05, respectively), whereas all aspects taken together are considered significantly more relevant in the group over 50 years of age (78.8% vs. 68.3%, *p*-value < 0.001). 

In the time window between 4 and 6 completed months of age, the participants were asked in which circumstances they would anticipate or postpone CF, as shown in Table 1.

### 3.3. CF Models

Regarding TW, 293/707 (41.4%) considered this approach to be characterized by food reduced to cream/puree, with the first meal being cream of rice/corn/tapioca, vegetables, meat or fish, and cheese, while 158/707 (22.3%) excluded the use of cheese and 174/707 (24.6%) excluded the use of both cheese and meat or fish. At the beginning of the CF period, 142/707 (20.1%) considered TW as the use of baby food made of fruit and by 44/707 (6.22%) as the introduction of food in small pieces according to what is consumed by other family members. Analyzing the responses stratified by age of the participants, there was a significantly greater tendency to consider TW as the use of food reduced into cream/puree and offering the first meal with cream of rice/corn/tapioca and vegetables (29.1% vs. 15.8%, *p*-value < 0.001) among those over 50 years, while among those under 50 years of age had a greater tendency to also consider the addition of meat, fish, and cheese (52.08% vs. 35.97%, *p*-value < 0.001). Furthermore, 313/707 (44.3%) of the PCPs stated that they recommend the use of BFs, whereas most of them 394/707 (55.7%) declared that they do not recommend them. However, this trend was reversed when the age of the participants was considered, as there was a greater statistically significant tendency to recommend baby food among those over 50 years of age (51.6% vs. 30.0% *p*-value < 0.001).

Overall, 55.7% (394/707) recommended fruit or fruit-derived products, 49.1% (347/707) cereals or cereal-derived products, 40.0% (283/707) vegetables or vegetable-derived products, 16.8% meat or meat-derived products, and finally 8.8% (62/707) fish or fish-derived products. The latter type of food was significantly more recommended among those under 50 years of age (13.7% vs. 6.2%, *p*-value < 0.01).

CF based on vegetarian or plant-based diets was routinely supported by 45/707 (6.1%) PCPs, only if planned by a specialist by 253/707 (35.8%), and only if expressly requested by caregivers by 257/707 (36.3%). Conversely, 180/707 (25.5%) declared that they do not support them in any case. Analysis of the age-stratified data showed a greater tendency to support such diets among those under 50 years of age; 41.67% vs. 32.76% (*p*-value < 0.05) and 10.8% vs. 3.6% (*p*-value < 0.001) supported them only if planned by a specialist and support in every case, respectively. This trend was confirmed by the opposition to their support in every case as expressed by 30.8% of those older than 50 years old vs. 15.0% of those younger than 50 years old (*p*-value < 0.001). CF based on the vegan diet was supported in any case by 9/707 (1.3%), only if planned by a specialist by 189/707 (26.7%), and only if expressly requested by the caregivers by 185/707 (26.2%). On the contrary, 342/707 (48.4%) stated that they do not support it in any case. Younger PCPs showed a greater tendency to support alternative dietary regimes. Among PCPs under 50 years of age, such a regimen was routinely supported by 2.9% vs. 0.4% of those older than 50 years of age (*p*-value < 0.01), only in the case of diets planned by a specialist by 32.9% vs. 23.5% (*p*-value < 0.01), and only if expressly requested by caregivers by 30.8% vs. 23.8% (*p*-value <0.05). On the other hand, 55.0% of participants older than 50 years of age vs. 35.4% of participants younger than 50 years old tended not to support them at all (*p*-value < 0.001).

In case of CF based on a vegan diet, supplementation with vitamin B12, folic acid, and docosahexaenoic acid (DHA) was recommended by 8.9% (63/707) of participants, while 42.0% (297/707) also recommended iron supplementation. In contrast, supplementation with vitamin B12 and, in case of deficiency, with folic acid alone or with folic acid, DHA, and iron was recommended by 8.8% (62/707) and 38.05% (269/707), respectively. Finally, 2.4% (17/707) stated that they do not recommend any supplementation. Comparing the data from the groups tested, supplementation with vitamin B12, folic acid, DHA, and iron was significantly more recommended among those over 50 years of age (46.5% vs. 33.3%, *p*-value < 0.01), while supplementation with vitamin B12 and eventually folic acid, DHA, and iron in the case of deficiency was significantly more recommended among those under 50 years of age (46.7% vs. 33.6%, *p*-value < 0.01).

Analyzing PCPs’ knowledge of the alternative approaches of BLW and ODCF and also TW, 297/707 (42.0%) declared to be familiar with both alternative CF approaches, 372/707 (52.6%) declared that they are only familiar with the ODCF approach, 38/707 (5.4%) only with the BLW approach, and 44/707 (6.22%) with none of them. Furthermore, when the results were stratified according to the age of the participants, 52.5% (126/240) of those who were younger than 50 years old and 36.6% (171/467) of those who were over 50 years old stated that they knew both alternative CF approaches (*p*-value < 0.001). The knowledge of using only ODCF also appeared to be more common among those over 50 (262/467, 56.1%) than among those under 50 (110/240, 45.8%) (*p*-value < 0.05), while BLW alone is not very popular in both groups (13/240, 5.4% vs. 25/467, 5.3%). Finally, the popularity of ODCF and BLW seemed to decrease significantly with the age of the participants, as 2.1% (5/240) of those with less than 50 years of age and 8.3% (39/467) of those with more than 50 years of age reported not knowing either method (*p*-value < 0.01).

The preferred CF methods recommended by our survey participants, also taking into consideration the parents’/caregivers’ requests, are summarized in Table 2.

### 3.4. Method of Parents’ or Caregivers’ Instructions

Overall, 89.7% of the sample (634/707) stated that they always discuss about CF with caregivers, 5.5% (39/707) only in the case of clinical problems highlighted during medical examinations, 3.7% (26/707) only if requested by caregivers, and 1.7% (12/707) of them said they do not give any information at all. The analysis of the data stratified by age showed a greater tendency to always give information (93.7% vs. 97.6%, *p*-value < 0.05) among those under the age of 50, whereas among those over the age of 50, there was a greater tendency to have such a discussion only in case of the occurrence of clinical problems (7.1% vs. 2.5%, *p*-value < 0.05) or not to have it at all (2.4% vs. 0.42%, *p*-value < 0.05). Moreover, 51.9% (367/707) of the PCPs interviewed stated that they always provide CF information in a pre-printed form, 37.2% (263/707) that they always provide it in a customized form, 8.9% (63/707) only if requested by caregivers, 3.0% (21/707) only in the case of critical issues highlighted during medical examinations, and 5.2% (37/707) said that they do not provide information at all. Analysis of the data stratified by the age of the participants showed a greater tendency to administer customized schemes and written information among those over 50 years of age (44.5% vs. 22.9%, *p*-value < 0.001), whereas those under 50 years of age showed a greater tendency to administer pre-printed forms (61.2% vs. 47.1%, *p*-value < 0.001) and to provide written schemes and information only in the case of critical issues highlighted during medical examinations (5.0% vs. 1.9%, *p*-value < 0.05). Analysis of the other responses showed no significant differences between the two age groups.

When approaching CF in infants of foreign origin, 76.1% of participants (538/707) claimed to mediate and discuss information on solid foods introduction with caregivers, 20.2% (143/707) set CF according to the customs of the infant’s family traditions and culture, and 11.5% (81/707) according to their local customs. There were no significant differences when comparing the results of the two groups.

### 3.5. Allergenic Food and CF

Analyzing the timing of allergenic foods introduction in CF, participants were asked about the suggested timing of the introduction of some frequently consumed allergenic foods. The timeframes considered by PCPs as appropriate for cow’s milk, eggs, gluten, fish, nuts, and soy introduction into children’s diets are shown in Table 3.

Suggested timings of the introduction of egg and fish in case of positive family history of food allergy are shown in Table 4.

## 4. Discussion

To our knowledge, our survey collects knowledge and approach to CF among the largest number of Italian PCPs so far, with a prevalence of female pediatricians. According to a report published by the Italian Ministry of Health in August 2023, 68.6% of PCPs working in Italy in 2021 were females [28]. This finding is in line with our sample, which showed a higher participation of female than male subjects (79.5% vs. 20.1%, *p*-value < 0.001). Considering the age of the participants, there is an increasing gradient across different age groups. This trend was expected, considering studies carried out regarding the socio-epidemiological trends about those employed by the Italian National Healthcare System over the years. Although female subjects accounted for the largest proportion among the groups over and under 50 years of age, there was a higher proportion of them among participants under 50 years of age (90.0% vs. 74.1%). This increasing “feminization” of the medical profession, with a larger presence of women in the younger classes, is in line with the data reported by the Italian Ministry of Health [28].

Concerning informative sources on CF, less than half of the participants declared to have learned about CF during their university studies. Nutrition is a fundamental milestone in infants and children’s lives, especially during the so-called “critical windows”, such as CF [4]. Pediatric nutritional issues should be taught in all medical university courses, both from a theoretical and a practical point of view. 

All the participants in our survey declared that CF should be started between 4 and 6 completed months of age, in line with the current national and international consensus documents [1,5,6,7]. In this timeframe, most of them decide to anticipate CF in case of growth deceleration. However, if infants with growth deceleration are divided into breastfed and formula-fed, only 11.2% of participants considered anticipating CF in exclusively breastfed infants with growth curve deflection, and most of them preferred offering formula milk instead of CFs, even if adequate neurodevelopmental milestones to start CF have been reached. We think this a fundamental issue to work on, as exclusively breastfed infants who have a growth deceleration without any other clinical reasons, having reached adequate for age neurodevelopmental milestones, should start CF instead of formula milk, as CF can provide adequate proteins, iron, and zinc, whose content in human milk may not be able to cover infants’ increasing needs from around 6 months of age onwards. In an online survey conducted in 2020 among 350 Italian PCPs, 8% of participants recommended CF start after 6 completed months of age [29]; in this sense, PCPs seem to have improved their attitude towards CF timing. In a more recent survey conducted by Congiu et al. on 595 Italian PCPs, 1% of participants recommended CF start beyond six completed months of age, and this result is consistent with the findings of our survey [26]. In 2017, a cross-sectional survey analyzed pediatricians’ practices about growth and nutrition for children under 2 years in a cohort of 698 American Academy of Pediatrics members: 2.3% of participants recommended solid foods introduction after six completed months of age, 4.2% after eight months of age, and 0.4% before 4 months of age [30].

Greater attention is needed when dealing with families of foreign origins: three out of four participants reported discussing and mediating a CF scheme and instruction with the family members and caregivers, respecting their tradition and culture.

TW was defined by most of the participants as food proposed as crème/puree, and 20.8% of them believed that the first food to be offered to infants should be fruit. This finding is not unexpected, as fruit puree or baby food based on fruit is deeply rooted in Italian culture as the first solid foods proposed to infants; thus, even if cereal, vegetables, and meat should be the first CFs to be introduced, this habit seems hard to abandon. Most of the participants did not recommend baby foods, even if this trend was not confirmed among those older than 50 years old, who preferred baby food to natural food items. This finding may have two different explanations: On the one hand, older PCPs tend to be more conservative and promote baby food consumption; on the other hand, they may be more aware of possible food safety issues related to non-baby food used in infants younger than 3 years old. Dembinskj et al. conducted a survey on CF practices on a sample of 303 Polish pediatricians working both in hospitals and in outpatients clinics, and they reported different results on this topic, as 51.8% of participants recommended commercial baby foods. They justified their choice based on the quality, known composition, and safety of commercial products compared to home-made preparations [31]. Non-traditional CF models were known by less than half of the participants of our survey, with a better knowledge among the younger ones. 

Vegetarian CF was not supported by one out of four of our study participants, even if those younger than 50 years of age tended to be more supportive of plant-based CF but only if planned by a specialist in pediatric nutrition. PCPs who support vegan CF are not homogenous on vitamin supplementation recommendations for these infants, and 2.4% of them did not recommend vitamins at all, even if vitamin B12 supplementation is mandatory when following a vegan diet. A vegan diet for infants is a controversial issue, and it is not advised by most of the current consensus papers [6,7]. However, pediatricians should support and respect the family’s decision without prejudices, aiming for the infant’s growth and neurodevelopment; hence, referral to a pediatric nutritional center might be advisable, when necessary.

The timing of allergenic foods introduction in CF was debated among our study participants: 7.9% of them considered introducing cow’s milk before 12 completed months of age. Most participants advised introducing eggs and fish before 12 months of age, even if 13.3% preferred to wait 12 months in case of a positive family history of food allergy. When it comes to dried fruits, 45.4% preferred to introduce them after 12 months of age, with a statistically significant difference between PCPs younger and older than 50 years (22.1% versus 57.4%). The tendency to delay dried fruit introduction may be due to its not usually wide consumption in Italy compared to other countries such as Australia or the USA, so on the one hand, PCPs may be less confident in dealing with this kind of food, and on the other hand, families may not be used to frequently consuming them. The AAP recommends waiting 3 to 5 days between the introduction of each new complementary food in an infant’s diet [32]. Samady et al. published the results of a survey involving 604 American pediatric health care professionals on recommendations on the age of CF introduction and waiting periods between the introduction of new foods: They found that only 66.3% of participants recommended waiting that time interval in case of infants at risk of food allergy and 38.6% in infants not at risk for food allergy [33]. Vassilopolu et al. investigated Greek PCPs’ attitudes towards allergenic foods introduction in CF in a sample of 233 pediatricians. They found that pediatricians with less than 15 years of practice often introduced allergenic foods earlier for high-risk infants compared to those with longer practice experience; they also highlighted a tendency to delay the introduction of common food allergens and to recommend longer intervals between each new food introduction, particularly for high-risk infants, in the whole cohort analyzed [34]. These results are consistent with those of our survey.

Our survey has some limitations. First, the questionnaire was filled out voluntarily, and this may generate a selection bias. Secondly, we did not ask about the region where the PCPs operate; therefore, we are not able to detect geographical differences in CF habits and attitudes. On the other hand, one of the main strengths of our study is that, to our knowledge, this is the largest study involving Italian PCPs on this topic. Moreover, we were able to divide our responders into two categories according to their age, therefore obtaining information on younger and older PCPs. Another important strength of our study is that we analyzed various aspects of CF, from the type (traditional or non-traditional) to the attitudes towards allergenic food introduction and plant-based CF.

## 5. Conclusions

The results of our survey show that PCPs in Italy are interested in CF. In our sample, most PCPs knew the correct timing for starting CF, but 13.3% of them tended to postpone commonly consumed allergenic food introduction (such as eggs and fish), with a higher percentage postponing the introduction of dried fruits. Moreover, only approximately half of them knew and supported non-traditional-type CF or plant-based CF, even if this tendency was greater among younger PCPs. CF is a very important and delicate nutritional and educational window for the infant and the whole family, so fundamentally, PCPs should guide a family’s dietary choices without prejudices, aiming for the infant’s growth and neurodevelopment while respecting each family’s tradition, culture, and beliefs. To our knowledge, our survey is the first to analyze PCPs’ attitude towards CF in clinical practice, focusing on multiple aspects. We analyzed timing of the introduction of solid foods, recommendations regarding allergenic foods introduction, attitudes toward traditional and non-traditional CF models, and the practical approach when interacting with families. We are the first to specifically focus on vegan and vegetarian CF and on PCPs’ attitude towards these emerging dietary patterns. Our sample is the largest recruited in the past decades, with the highest response rate registered so far. To our knowledge, our survey is the first to give an overall, comprehensive picture of the current attitude towards CF of a considerable percentage of Italian PCPs.

Although to our knowledge, this is the first study examining PCPs’ attitudes on CF, in which so many different items were addressed, further studies are needed to deepen our knowledge on the real-life habits and behaviors concerning this important nutritional issue, with the aim to plan educational programs according to the latest recommendations on healthy infants’ nutrition. 

## Figures and Tables

**Table 1:** Circumstances in which the start of CF should be anticipated or postponed in the time window between 4 and 6 completed months of age, according to PCPs.

Questionnaire Items	Total Number *n* = 707	<50 Years Old *n* = 240	≥50 Years Old *n* = 467	*p*-Value
In the time window between 4 and 6 completed months of age, when do you consider it appropriate to anticipate the start of complementary feeding? (multiple-choice question)				
In the case of exclusive breastfeeding and if a significant deviation of the stature–ponderal curve is observed (having reached the neurodevelopmental stages necessary for its initiation)	80 (11.32%)	22 (9.17%)	58 (12.42%)	0.20
If exclusively formula milk is used and if a significant deviation of the stature–ponderal curve is observed (having reached the neurodevelopmental stages necessary for its initiation)	85 (12.02%)	38 (15.83%)	47 (10.06%)	<0.05
In the case of mixed-mode feeding (breastfeeding + formula milk) and if a significant deviation of the stature–ponderal curve is observed (having reached the neurodevelopmental stages necessary for its initiation)	56 (7.92%)	20 (8.33%)	36 (7.71%)	0.77
In none of these cases	140 (19.80%)	51 (21.25%)	89 (19.06%)	0.49
In all these cases	411 (58.13%)	130 (54.17%)	281 (60.17%)	0.13
Considering the time window of 4 to 6 completed months of age, what aspects do you consider in postponing the start of complementary feeding? (multiple-choice question)				
Adequate human breast milk intake	355 (50.21%)	115 (47.92%)	240 (51.39%)	0.38
Stature–ponderal curve without deflections	383 (54.17%)	147 (61.25%)	236 (50.54%)	<0.01
Family history of allergy	37 (5.23%)	6 (2.50%)	31 (6.64%)	<0.05
Family request	194 (27.44%)	67 (27.92%)	127 (27.19%)	0.84

**Table 2:** The preferred method of CF initiation recommended by PCPs, the method most frequently requested by caregivers, and the percentage of caregivers requesting CF to be initiated as BLW and ODCF.

Questionnaire Items	Total Number *n* = 707	<50 Years Old *n* = 240	≥50 Years Old *n* = 467	*p*-Value
Which method do you usually recommend to caregivers to undertake complementary feeding? (multiple-choice question)				
Traditional weaning	336 (47.52%)	59 (24.58%)	277 (59.31%)	<0.001
Sweet milk flour	8 (1.13%)	3 (1.25%)	5 (1.07%)	0.83
Baby-led weaning	88 (12.45%)	45 (18.75%)	43 (9.21%)	<0.001
On-demand complementary feeding	232 (32.81%)	112 (46.67%)	120 (25.70%)	<0.001
Traditional spoon-feeding with adult food tastings	290 (41.02%)	132 (55.00%)	158 (33.83%)	<0.001
Based on your clinical experience, which approach to complementary feeding is most often requested by caregivers? (multiple-choice question)				
Traditional weaning	391 (55.30%)	109 (45.42%)	282 (60.39%)	<0.001
Sweet milk flour	15 (2.12%)	3 (1.25%)	12 (2.57%)	0.19
Baby-led weaning	29 (4.10%)	14 (5.83%)	15 (3.21%)	0.07
On-demand complementary feeding	149 (21.07%)	65 (27.08%)	84 (17.99%)	<0.05
Traditional spoon-feeding with adult food tastings	283 (40.03%)	119 (49.58%)	164 (35.12%)	<0.001
Which percentage of caregivers request to undertake complementary feeding of their child with “baby-led weaning” or “on-demand complementary feeding”?				
<10%	343 (48.51%)	74 (30.83%)	269 (57.60%)	<0.001
10–25%	192 (27.16%)	84 (35.00%)	108 (23.13%)	<0.01
25–50%	77 (10.89%)	38 (15.83%)	39 (8.35%)	<0.05
50–75%	67 (9.48%)	36 (15.00%)	31 (6.64%)	<0.01
75–100%	28 (3.96%)	8 (3.33%)	20 (4.28%)	0.54

**Table 3:** Timeframes considered by PCPs as appropriate for cow’s milk, eggs, gluten, fish, nuts, and soya introduction into children’s diets.

Questionnaire Items	Total Number *n* = 707	<50 Years Old *n* = 240	≥50 Years Old *n* = 467	*p*-Value
In your opinion, at what age is it appropriate to introduce cow’s milk into a child’s diet? (multiple-choice question)				
From the beginning of the complementary feeding period	6 (0.85%)	2 (0.83%)	4 (0.86%)	0.67
Between 9 and 12 months of age	50 (7.07%)	12 (5.00%)	38 (8.14%)	0.12
1–2 years old	499 (70.58%)	209 (87.08%)	290 (62.10%)	<0.001
2–3 years old	102 (14.43%)	18 (7.50%)	84 (17.99%)	<0.001
Beyond 3 years old	56 (7.92%)	3 (1.25%)	53 (11.35%)	<0.001
In your opinion, at what age is it appropriate to introduce eggs into a child’s diet? (multiple-choice question)				
4–6 months of age	115 (16.27%)	52 (21.67%)	63 (13.49%)	<0.01
6–7 months of age	311 (43.99%)	149 (62.08%)	162 (34.69%)	<0.001
7–8 months of age	190 (26.87%)	35 (14.58%)	155 (33.19%)	<0.001
11–12 months of age	94 (13.30%)	16 (6.67%)	78 (16.70%)	<0.001
After 12 months of age	21 (2.97%)	4 (1.67%)	17 (3.64%)	0.14
In your opinion, at what age is it appropriate to introduce gluten into a child’s diet? (multiple-choice question)				
4–6 months of age	227 (32.11%)	85 (35.42%)	142 (30.41%)	0.18
6–7 months of age	475 (67.19%)	161 (67.08%)	314 (67.24%)	0.97
7–8 months of age	17 (2.40%)	4 (1.67%)	13 (2.78%)	0.26
11–12 months of age	3 (0.42%)	1 (0.42%)	2 (0.43%)	0.73
After 12 months of age	3 (0.42%)	2 (0.83%)	1 (0.21%)	0.27
In your opinion, at what age is it appropriate to introduce fish into a child’s diet? (multiple-choice question)				
4–6 months of age	152 (21.50%)	71 (29.58%)	81 (17.34%)	<0.001
6–7 months of age	387 (54.74%)	154 (64.17%)	233 (49.89%)	<0.001
7–8 months of age	160 (22.63%)	26 (10.83%)	134 (28.69%)	<0.001
11–12 months of age	26 (3.68%)	3 (1.25%)	23 (4.93%)	<0.05
After 12 months of age	5 (0.71%)	1 (0.42%)	4 (0.86%)	0.51
In your opinion, at what age is it appropriate to introduce dried fruit into a child’s diet? (multiple-choice question)				
4–6 months of age	104 (14.71%)	52 (21.67%)	52 (11.13%)	<0.001
6–7 months of age	195 (27.58%)	114 (47.50%)	81 (17.34%)	<0.001
7–8 months of age	51 (7.21%)	20 (8.33%)	31 (6.64%)	0.41
11–12 months of age	53 (7.50%)	13 (5.42%)	40 (8.57%)	0.13
Beyond 12 months of age	321 (45.40%)	53 (22.08%)	268 (57.39%)	<0.001
In your opinion, at what age is it appropriate to introduce soy into a child’s diet? (multiple-choice question)				
4–6 months of age	160 (22.63%)	58 (24.17%)	102 (21.84%)	0.48
6–7 months of age	270 (38.19%)	129 (53.75%)	141 (30.19%)	<0.001
7–8 months of age	68 (9.62%)	20 (8.33%)	48 (10.28%)	0.41
11–12 months of age	48 (6.79%)	12 (5.00%)	36 (7.71%)	0.18
Beyond 12 months of age	181 (25.60%)	37 (15.42%)	144 (30.84%)	<0.001

**Table 4:** Suggested timing of introduction of egg and fish in case of positive family history of food allergy.

Questionnaire Items	Total Number *n* = 707	<50 Years Old *n* = 240	≥50 Years Old *n* = 467	*p*-Value
In children with a family history of food allergy, what recommendations do you provide regarding the introduction of eggs and fish? (multiple-choice question)				
Beyond 12 months of age	94 (13.30%)	13 (5.42%)	81 (17.34%)	<0.001
From 4 months of age, preferably during breastfeeding	62 (8.77%)	16 (6.67%)	46 (9.85%)	0.16
From 6 months of age, preferably during breastfeeding	290 (41.02%)	116 (48.33%)	174 (37.26%)	<0.01
From 6 months of age	549 (77.65%)	212 (88.33%)	337 (72.16%)	<0.001
I recommend avoiding these foods and I suggest an allergological examination	11 (1.56%)	2 (0.83%)	9 (1.93%)	0.35

## Data Availability

The data presented in this study are available on request from the corresponding author.

## Abbreviations

AAND	American Academy of Nutrition and Dietetics
AAP	American Academy of Pediatrics
AHA	American Heart Association
BLW	baby-led weaning
BF	breastfeeding
BFs	baby foods
CD	celiac disease
CF	complementary feeding
CFs	complementary foods
DHA	docosahexaenoic acid
ESPGHAN	European Society of Paediatric Gastroenterology, Hepatology and Nutrition
FA	food allergy
FIMP	Federazione Italiana Medici Pediatri
HBM	human breast milk
ODCF	on-demand complementary feeding
PBD	plant-based diet
PCP	primary care pediatrician
SIDOHaD	Società Italiana per lo Sviluppo e le Origine della Salute e delle Malattie
SINUPE	Società Italiana di Nutrizione Pediatrica
SIPPS	Società Italiana di Pediatria Preventiva e Sociale
SW	self-weaning
TW	traditional weaning
UNICEF	United Nations Children’s Fund
WHO	World Health Organization

## Appendix A

**Complementary Feeding Survey** **We Kindly Ask You to Answer the Following Questions.** **(You Can Indicate More Than One Answer per Question)** *** It is a Mandatory Question.**
Gender *: ☐ Male ☐ Female ☐ I prefer not to answer
Age *: ☐ <30 years old ☐ 30–40 years old ☐ 40–50 years old ☐ 50–60 years old ☐ >60 years old
What is your specialization? *: ☐ Pediatrics ☐ Other
During which occasion did you learn useful notions that you use for the setting up of a complementary feeding? *: ☐ During university studies ☐ Specific refresh courses ☐ Scientific journals ☐ Guidelines/position papers ☐ Empirical methodology
In the time window between 4 and 6 completed months of age, when do you consider it appropriate to anticipate the start of complementary feeding? *: ☐ In the case of exclusive breastfeeding and if a significant deviation of the stature–ponderal curve is observed (having reached the neurodevelopmental stages necessary for its initiation) ☐ If exclusively formula milk is used and if a significant deviation of the stature–ponderal curve is observed (having reached the neurodevelopmental stages necessary for its initiation) ☐ In the case of mixed-mode feeding (breastfeeding + formula milk) and if a significant deviation of the stature–ponderal curve is observed (having reached the neurodevelopmental stages necessary for its initiation) ☐ In none of these cases ☐ In all these cases
Considering the time window of 4 to 6 completed months of life, what aspects do you consider in postponing the start of complementary feeding? *: ☐ Adequate human breast milk intake ☐ Stature–ponderal curve without deflections ☐ Family history of allergy ☐ Family request
Are you aware of the complementary feeding methods called “baby-led weaning” and “on-demand complementary feeding”? *: ☐ I am aware of the methodology defined as “baby-led weaning”. ☐ I am aware of the methodology defined as “on-demand complementary feeding” ☐ No, neither of them ☐ Yes, both of them
What do you mean by “traditional weaning”? *: ☐ Complementary feeding with food reduced into cream/puree, offering the first meal with cream of rice/corn/tapioca and vegetables ☐ Complementary feeding with food reduced into cream/puree, offering the first meal with cream of rice/corn/tapioca, vegetables, and meat or fish ☐ Complementary feeding with food reduced into cream/puree, offering the first meal with cream of rice/corn/tapioca, vegetables, meat or fish, and cheese ☐ Complementary feeding with the initial introduction of baby food made of fruit ☐ Complementary feeding with the introduction of food in small pieces, according to what is consumed by other family members
If you are aware, what do you mean by “on-demand complementary feeding” and “baby-led weaning”? *: ☐ Alternative methods of complementary feeding in which the child eats alone with the use of a spoon/fork the food in the original form prepared by the parents ☐ Alternative methods of complementary feeding in which the child decides which and how many solid foods to eat in the form of finger foods, sitting at the table with the family ☐ Alternative complementary feeding methods in which the child decides how much of the “traditional weaning” foods to eat at the time of day he wishes ☐ Alternative methods of complementary feeding in which the child, in addition to taking baby foods and/or pureed foods, occasionally takes foods reduced to small pieces
Which method do you usually recommend to caregivers to undertake complementary feeding? *: ☐ Traditional weaning ☐ Sweet milk flour ☐ Baby-led weaning ☐ On-demand complementary feeding ☐ Traditional spoon-feeding with adult food tastings
Based on your clinical experience, which approach to complementary feeding is most often requested by caregivers? *: ☐ Traditional weaning ☐ Sweet milk flour ☐ Baby-led weaning ☐ On-demand complementary feeding ☐ Traditional spoon-feeding with adult food tastings
Which percentage of caregivers request to undertake complementary feeding of their child with “baby-led weaning” or “on-demand complementary feeding”? *: ☐ <10% ☐ 10–25% ☐ 25–50% ☐ 50–75% ☐ 75–100%
At how many months of age do you usually recommend the start of complementary feeding in the case of exclusive breastfeeding? *: ☐ Before 4 months of age ☐ 4–6 months of age ☐ After 7 months of age
At how many months of age do you usually recommend the start of complementary feeding in the case of exclusive use of formula milk? *: ☐ Before 4 months of age ☐ 4–6 months of age ☐ After 7 months of age
At how many months of age do you usually recommend the start of complementary feeding in the case of mixed-mode feeding (breastfeeding + formula milk) ☐ Before 4 months of age ☐ 4–6 months of age ☐ After 7 months of age
What leads you to the decision to start complementary feeding? *: ☐ Reaching neurodevelopmental milestones ☐ Nutritional needs ☐ Family compliance ☐ Weight gain of the child ☐ All the above cases
Which aspects do you consider anticipating the start of complementary feeding? *: ☐ Type of feeding performed in previous months ☐ Weight-related growth curve changes judged inadequate ☐ Infant disorders (e.g., GERD) ☐ Request by the family ☐ Risk of nutritional deficiencies (e.g., iron)
In the case of patients of foreign ethnicity, which schemes do you use to undertake complementary feeding? *: ☐ According to local customs ☐ According to the customs of the respective culture ☐ Seeking discussion and mediation with caregivers and their customs
Do you usually discuss with caregivers on which recommendations to follow regarding complementary feeding and its administration? *: ☐ Yes, always ☐ Yes, if requested by caregivers ☐ Yes, but only in the case of criticalities highlighted during medical examinations ☐ No
Do you provide schemes and written information to families about complementary feeding? *: ☐ Yes, always, customized ☐ Yes, always, pre-printed ☐ Yes, if requested by caregivers ☐ Yes, but only in the case of critical issues highlighted during medical examinations ☐ No
Do you usually recommend the use of “baby foods”? *: ☐ Yes ☐ No
Do the parents of the children you assist generally adhere to the recommendations you give to them? *: ☐ Yes, completely (100%) ☐ Almost completely (75–100%) ☐ The majority (50–75%) ☐ A minority (25–50%) ☐ Usually not (0–25%)
Do you support complementary feeding based on vegetarian or plant-based diets? *: ☐ Yes, in any case ☐ Yes, but only if diets are planned by a specialist ☐ No, in any case ☐ No, only if expressly requested by caregivers
Do you support complementary feeding based on a vegan diet? *: ☐ Yes, in any case ☐ Yes, but only if diets are planned by a specialist ☐ No, in any case ☐ No, only if expressly requested by caregivers
In the case of complementary feeding based on a vegan diet, which additional supplements do you consider necessary (in addition to those usually recommended for children receiving an omnivorous diet)? *: ☐ Vitamin B12, folic acid, and docosahexaenoic acid ☐ Vitamin B12, folic acid, docosahexaenoic acid, and iron ☐ Vitamin B12 and, in case of deficiency, folic acid, docosahexaenoic acid, and iron ☐ Vitamin B12 and, in case of deficiency, folic acid ☐ No additional supplementation
What is the first kind of food you recommend starting complementary feeding routinely? *: ☐ Cereals or cereal-derived products ☐ Meat or meat-derived products ☐ Fruit or fruit-derived products ☐ Fish or fish-derived products ☐ Vegetables or vegetable-derived products
In children with a family history of food allergy, what recommendations do you provide regarding the introduction of eggs and fish? *: ☐ Beyond 12 months of age ☐ From 4 months of age, preferably during breastfeeding ☐ From 6 months of age, preferably during breastfeeding ☐ From 6 months of age ☐ I recommend avoiding these foods and I suggest an allergological examination
In your opinion, at what age it is possible to add salt to foods and/or food preparations into a child’s diet? (healthy subjects) *: ☐ From the beginning of the complementary feeding period ☐ Between 9 and 12 months of age ☐ 1–2 years old ☐ 2–3 years old ☐ Beyond 3 years old
In your opinion, at what age it is possible to add sugar to foods and/or food preparations into a child’s diet? (healthy subjects) *: ☐ From the beginning of the complementary feeding period ☐ Between 9 and 12 months of age ☐ 1–2 years old ☐ 2–3 years old ☐ Beyond 3 years old
In your opinion, at what age is it appropriate to introduce cow’s milk into a child’s diet? ☐ From the beginning of the complementary feeding period ☐ Between 9 and 12 months of age ☐ 1–2 years old ☐ 2–3 years old ☐ Beyond 3 years old
In your opinion, at what age is it appropriate to introduce eggs into a child’s diet? ☐ 4–6 months of age ☐ 6–7 months of age ☐ 7–8 months of age ☐ 11–12 months of age ☐ Beyond 12 months of age
In your opinion, at what age is it appropriate to introduce gluten into a child’s diet? ☐ 4–6 months of age ☐ 6–7 months of age ☐ 7–8 months of age ☐ 11–12 months of age ☐ Beyond 12 months of age
In your opinion, at what age is it appropriate to introduce fish into a child’s diet? ☐ 4–6 months of age ☐ 6–7 months of age ☐ 7–8 months of age ☐ 11–12 months of age ☐ Beyond 12 months of age
In your opinion, at what age is it appropriate to introduce dried fruit into a child’s diet? ☐ 4–6 months of age ☐ 6–7 months of age ☐ 7–8 months of age ☐ 11–12 months of age ☐ Beyond 12 months of age
In your opinion, at what age is it appropriate to introduce soy into a child’s diet? ☐ 4–6 months of age ☐ 6–7 months of age ☐ 7–8 months of age ☐ 11–12 months of age ☐ Beyond 12 months of age
